# Anti-cancer properties of chitosan /*Lactobacillus acidophilus* secretome nanoparticle on signaling pathways of colorectal cancer in colon adenocarcinoma (Caco-2) cell line

**DOI:** 10.1186/s12885-025-14315-5

**Published:** 2025-06-02

**Authors:** Masoumeh Saberpour, Rahimeh Maqsoodi, Bita Bakhshi

**Affiliations:** Department of Bacteriology, Faculty of Medical Sciences, Tarbiat Modares University, Jalal-Ale-Ahmad Ave, Tehran, 14117-13116 Iran

**Keywords:** Anti-cancer, Chitosan nanoparticle, Colorectal cancer, *Lactobacillus acidophilus*, Signaling pathways

## Abstract

**Background:**

Colorectal cancer (CRC) has emerged as a global health concern, as evidenced by its position as the second leading cause of cancer-related mortality. This underscores the necessity for effective disease management strategies. The present study aims to assess the impact of chitosan nanoparticles (CSNP) conjugated with the *Lactobacillus acidophilus* secretome (CSNP/L.a-sup), on the signaling pathways associated with CRC.

**Methods:**

The CSNP/L.a-sup was prepared using an ionic gelation procedure, and its particle size, surface charge, and morphology were evaluated using dynamic light scattering, zeta potential, and scanning electron microscopy. The encapsulation efficiency (EE) and the protein released from CSNP/L.a-sup were assayed using a BCA assay kit. CSNP/L.a-sup toxicity on colon adenocarcinoma (Caco-2) and human dermal fibroblasts (HDF) cells was assessed via the MTT assay. The expression levels of CRC signaling pathway genes were examined using real-time polymerase chain reaction (PCR).

**Results:**

The size of CSNP/L.a-sup was detected at 478.6 ± 219.9 nm, with a surface charge of -8.9 mV. The protein released from CSNP/L.a-sup was observed 76% at pH ~ 6.8 after 48 h, with EE of 74.6%. The viability of Caco-2 and HDF cells against CSNP/L.a-sup was found to be 85.5% and 92.6%, respectively. The uptake of CSNP/L.a-sup by Caco-2 cells occurs in a time-dependent manner, with initial absorption observed within 1 h and substantial internalization achieved after 3 h. CSNP/L.a-sup led to a significant decrease in the expression of *β-Catenin*, *TGF-α*, and *TGF-β* genes, with respective changes of 0.42, 0.79, and 0.16-fold. In contrast, CSNP/L.a-sup led to a significant increase in the expression of *PTEN* and *caspase-9* suppressor genes, with changes of 42.1 and 114.3-fold, respectively. The inhibitory effect of CSNP/L.a-sup on *TGF-α* gene expression appears to be more closely associated with the CSNP compartment, while the enhancing effect of CSNP/L.a-sup on *PTEN* gene expression is linked to L.a-sup.

**Conclusion:**

This investigation signifies an inaugural exploration into the potential of a combination therapy comprising secretome of probiotic bacteria and chitosan nanostructures. This approach constitutes a substantial advancement in the field of developing efficacious treatment strategies, offering novel insights into the management of CRC.

## Introduction

Colorectal cancer (CRC) is one of the most important causes of mortality worldwide, responsible for thousands of deaths every year [1, 2]. In Iran, the incidence of CRC is increasing [3]. Under normal conditions, body cells are modulated by numerous genes, and when these genes become dysregulated, cancer occurs. Subsequently, in cancer, signaling pathways is disrupted, and leading to an overexpression of oncogenes and decreasing of the expression of tumor suppressors [4]. Several signaling pathways play the crucial role in the regulation of CRC, including the pathways Wnt/β-Catenin (*β-Catenin*) [5], Phosphatidylinositol 3-kinase (*PI3K*) [6], transforming growth factor (*TGF-β*), Epidermal growth factor receptor (EGFR) (*TGF-α*) [6], Notch (*HES1*, *PTEN*), Hedgehog (SHH) (*Bcl2*, *Gli2*) [7], and genes such as interleukin 6 (*IL-6*), Toll-like receptor 4 (*TLR4*) [8], and carcinoembryonic antigen (*CEA*) [9].

Considering that cancer treatments are associated with many side effects and may lead to a decrease in the life of patient, identifying new therapeutic agents is essential. Moreover, targeting signaling pathways opens a new insight for CRC treatment [10]. Recently, probiotics [11] and nanoparticles with anti-cancer properties have attracted the attention of researchers [12, 13]. Probiotics are beneficial organisms in the digestive tract, that have anti-inflammatory and anti-cancer properties [11, 14]. For instance, *Lactobacillus acidophilus* (*L. acidophilus*) is a probiotic with anti-tumor and anti-proliferative effects. In a study by Isazadeh et al. (2020), have demonstrated that *L. acidophilus* secretome can prevent the proliferation of colon adenocarcinoma (Caco-2) cell line by inducing apoptosis and increase the survival rate of CRC patients [15]. In another research has indicated that the combination of the supernatant of *Lactobacillus. plantarum* and 5-fluorouracil (5-FU) have a inhibitory effect on Wnt/β-Catenin signaling pathway [16].

Previous studies have shown that the anticancer effects of *L. acidophilus* are attributed to its cell free supernatant (CFS). CFS is containing a wide range of beneficial compounds by tumor suppressing properties [17], such as SlpA, SlpB, and SlpX protein that between these SlpA is a promising strategy for CRC treatment [18]. Probiotic metabolites and bioactive molecules are multifunctional agents with anti-microbial, anti-inflammatory, antioxidant, immunomodulatory, and anti-proliferative activities [19]. They can act as their parent live cells and are a safe alternative to live probiotic cells. However, bioactive macromolecules are less stable and ineffective than live bacteria. One of the major problems with potent anticancer drugs is ensuring they reach the target site through a suitable carrier. Therefore, choosing an effective drug carrier is crucial for achieving optimal anticancer treatment results.

Nano-delivery strategies can enhance how well therapeutic agents are spread around the body, reducing side effects and/or increasing how well they work. Making nanocarriers smaller, changing their shape and improving their surface properties can make delivery better [20]. In recent years, researchers have focused on chitosan as a unique drug delivery system for cancer treatment. Chitosan is an ideal carrier for anticancer drugs with appropriate properties, such as biodegradable, nontoxic, and suitable bio adhesion. Chitosan has a polycationic surface that facilitates hydrogen and ionic binding within the body [21]. It is attached to negatively charged mucosal membranes and open tight junctions between epithelial cells, which increases the residence time of drug at the target site, thereby enhancing cellular uptake anti-cancer drugs. Encapsulating of CFS with CSNP is not only one strategy to protect of CFS against enzymatic degradation but also it ensures the delivery of a larger amount of CFS to the target site, which can be an effective therapeutic approach combat to CRC [22]. In a study by Wu et al. (2024) have indicated that a novel formulation including poly-L-glutamic acid conjugated with 7-ethyl-10-hydroxycamptothecin, CSNP, and *Bifidobacterium bifidum* led to an successfully drug delivery, with enhancing drug concentration at the target site and facilitating cellular uptake in tumor cells [23]. Numerous studies have confirmed CSNP effectiveness on the expression of genes associated with CRC signaling pathways. Previous studies showed that CSNP has an inhibitory impact on the *IL6* gene expression and an inducing effect on the *PTEN* gene expression in breast [24], and lung [25] cancers. No previous study has evaluated the effect of a combination of chitosan and *L. acidophilus* cell free supernatant on a wide range of genes involved in CRC signaling pathways. The novelty of this study lies in the combination of the secretome of probiotic bacteria with chitosan nanostructures in the treatment of CRC, with the potential for enhanced delivery, synergistic therapeutic effects, targeted action, immune modulation, and reduced toxicity, all of which could significantly improve treatment outcomes for patients with colorectal cancer. Whilst the present study constitutes merely an inaugural investigation into the potential of a combinational therapy for CRC treatment, it is nevertheless a significant step towards the development of new and effective treatment strategies. So, this research designed to examine the effects of CSNP/L.a-sup on the expression of genes related to CRC signaling pathways with a more targeted approach.

## Materials and methods

### Growth conditions of *L. acidophillus*

*L. acidophillus* strain was obtained from the archives of the bacteriology department of Tarbiat Modares University of Medical Science (Tehran, Iran). *L. acidophillus* was cultured on a plate of contain de Man, Rogosa and Sharpe (MRS) agar medium (Merck, Germany), and incubated under anaerobic condition at 37 °C for 72 h using a gas pack (Merck, Germany). Following this time, a colony of *L. acidophillus* was isolated, inoculated into Brain Heart Infusion (BHI) broth medium (Merck, Germany), and incubated under anaerobic conditions at 37 °C for 24 h. The optical density (OD) of the bacterial suspension was measured using a spectrophotometer (WPA, England) at a wavelength of 620 nm. The OD of the bacterial suspension was found to be equivalent to the 0.5 McFarland Standard, with an OD_620_ reading of approximately 0.08. The preparation of CFS involved the centrifugation of the bacterial suspension at 3,000 rpm for 5 min, followed by the removal of bacterial cells using a 0.22-µm filter. To ensure the absence of bacterial cells in CFS, it was re-cultured in MRS agar medium under both aerobic and anaerobic conditions [26, 27].

### Preparation of CSNP/L.a-sup

The CSNP/L.a-sup was synthesized via an ionic gelation procedure. In summary, the preparation of CSNP involved the addition of 1500 µL of phosphate-buffered saline (PBS) (1X) to 500 µL of tripolyphosphate (TPP) at a ratio of 1:4. Subsequently, 5 mL of chitosan solution was transferred to the PBS-TPP mixture at a ratio of 2:5. The resulting mixture was stirred under sterile conditions at 25 °C for 1 h. The synthesis of CSNP/L.a-sup was accomplished by the dropwise addition of 500 µL of TPP to 1500 µL of the culture medium of *L. acidophilus* at a ratio of 1:4. The resulting mixture was subsequently added to 5 mL of the chitosan solution at a ratio of 2:5. The mixture was then stirred under gentle magnetic stirring at room temperature for 1 h. The pH of the resulting solution was meticulously adjusted to a range of 8–9 using 1 M NaOH and HCl. Subsequently, the Falcon containing the nanoparticles was subjected to a centrifugal process at 16,000 rpm at 4 °C for a duration of 30 min. Following the removal of the upper layer, the final pellet was subjected to lyophilization and stored as a nanoparticle at -20 °C for future use. The size, charge, and morphology properties of the nanoparticles were analyzed using dynamic light scattering (DLS), zeta potential (ZP), and scanning electron microscopy (SEM), respectively [28].

### Determination of loaded protein and entrapment efficiency (EE) of CSNP/L.a-sup

In the present study, the BCA kit was utilized to evaluate the total protein within L.a-sup and the unloaded protein within CSNP/L.a-sup following nanoparticle formation. To ascertain the loaded protein within CSNP/L.a-sup, the quantity of residual protein following nanoparticle formation was subtracted from the initial protein content in L.a-sup. The following formula was used to calculate the quantity of EE% of CSNP/L.a-sup [29].

$$\begin{aligned}\:Percentage\,of\,Entrapment\,&Efficiency\,\left({EE\,\% } \right)\,\\&\quad=\,a - b/a\, \times \,100\end{aligned}$$

a: The total protein within L.a-sup, b: Unloaded protein after CSNP/L.a-sup formation.

### The release of protein from CSNP/L.a-sup

The amount of released protein from CSNP/L.a-sup was assayed in the similar condition of (small intestine; pH ~ 6.8), (stomach; pH ~ 1.8), and (colon; pH ~ 7.4) in a time interval of 2, 6, 12, 24, and 48 h by BCA assay kit. In brief, 5 mL of sterile PBS (1X) was added to the falcon of contains CSNP/L.a-sup, and then the mixture (PBS + CSNP/L.a-sup) was stirred and incubated at 37 °C. To evaluate of the released protein in 2, 6, 12, 24, and 48 h of time interval, 25 µL of the mixture was extracted and replaced with 25 µL of sterile PBS (1X) to maintain the volume and condition [30]. To calculate the released protein from CSNP/L.a-sup the following formula was used.

$$Release \%\ =\,a/b\, \times \,100$$

a: Protein released at time interval, b: Loaded protein within CSNP/L.a-sup.

### Cellular uptake evaluation

The effectiveness of the designed nanoparticle is contingent upon cellular absorption. Cell membranes interact with CSNP, which possess a positive charge. Due to electrostatic interactions, negatively charged cancer cells exhibit a preference for the absorption of CSNP with a positive charge. Consequently, CSNP is regarded as an effective drug delivery system for penetrating the cell-free supernatant of *L. acidophilus* within Caco-2 cells [30]. The cellular uptake of CSNP/L.a-sup was determined by adding CSNP/L.a-sup to 1-(3-dimethylaminopropyl)-3-ethylcarbodiimide hydrochloride and N-hydroxy-succinimide in a ratio of 4:2:1. Following this addition, rhodamine B (Rh B) solution was supplemented to the nanoparticle solution. The mixture was then stirred for 18 h at room temperature. Finally, 40 µg of CSNP conjugated with Rh B was added to Caco2 cells and incubated for 2 h at room temperature. Following this time, the cells were examined with a fluorescent microscope [31, 32].

### Colon adenocarcinoma (Caco-2) and human dermal fibroblast (HDF) cells Preparation

The Caco-2 (as a cancer cell) and HDF (as a normal cell) cells were procured from the Pasteur Institute of Iran (Tehran, Iran). The cells were cultivated in a T75 flask (SPL, Korea) containing high-glucose Dulbecco’s Modified Eagle’s Medium (DMEM) (Sigma, Germany), which was enriched with 10% fetal bovine serum (FBS) (Gibco, USA), 1% penicillin-streptomycin (1X) (Gibco, USA), and 2% L-glutamine (DNA Biotech-Iran). Subsequently, the cells were placed in an incubator maintained at 37 °C with 5% CO2 for a duration of 48 h. Following this, the cells were monitored using an inverted microscope. When the cells reached 80–90% confluency, they were sub-cultured into a new flask [33].

### Cytotoxicity effects of CSNP/L.a-sup on Caco-2 and HDF cells

The viability of cells was assessed after exposure to CSNP, L.a sup, and CSNP/L.a-sup using MTT solution, including 3-(4, 5-dimethylthiazol-2-yl)-2, 5-diphenyl tetrazolium bromide assay (DNA Biotech, Iran). A total of 1 × 10^3^ cells were seeded in each well of a 96-well culture plate (SPL, Korea). The plate was then incubated at 37 °C with 5% CO2 for a period of 48 h. The specified concentrations of CSNP (0.05%), L.a sup (1000 µg), and CSNP/L.a-sup (1000 µg ± 0.05%) were utilized for exposure of the cells. Then the cells were incubated at 37 °C with 5% CO2 for 24 h. Subsequently, the culture medium was discarded from each well and replaced with 100 µL of MTT solution. The cells were then placed in a dark environment and incubated at 37 °C within 4 h. Finally, the MTT solution from each well was discarded and 100 µL of dimethyl sulfoxide (DMSO) (Sigma Aldrich, USA) was transferred to each well and mixed. In the final step, the optical density of each well was measured using an ELISA reader (800 TS, BioTek, Winooski, Vermont, USA) at a wavelength of 570 nm [34].

### Extraction of RNA and synthesize of complementary DNA (cDNA)

The RNA extraction process was carried out in accordance with the protocol outlined in the RNA Miniprep Super Kit (Bio Basic, Canada). To prepare a 6-well culture plate, 2 × 10^4^ cells were seeded in each well that contained 3 mL of DMEM enriched with 10% FBS, and incubated at 37 °C for 48 h. After Caco-2 cells reached 80% confluency, the culture medium was removed from each well and replaced with 3 mL of antibiotic-free DMEM. Subsequently, 150 µL of L.a-sup in a final concentration of 1000 µg was added to each well. In addition, specified concentrations of CSNP (0.05%) and CSNP/L.a-sup (1000 µg ± 0.05%) were dissolved within 150 µL of antibiotic-free DMEM and added to each well. Furthermore, 5-FU was employed as a control standard group for the evaluation of the efficacy of the designed nanostructure in regulating the signaling pathways associated with colorectal cancer. The plate was subsequently incubated at 37 °C with 5% CO2 for 24 h. The extraction of RNA was performed from both treated cells and untreated cells (as control). The final concentration of RNA was read at 260–280 nm. Finally, cDNA was synthesized using RNA at concentrations between 1.8 and 2 according to the Yekta Tajhiz Azma procedure from Iran.

### Assessment of effect of CSNP/L.a-sup on the expression of genes associated with CRC signaling pathways using real-time PCR

The effect of CSNP, L.a sup, and CSNP/L.a-sup on the expression of genes related to CRC signaling pathways including *β-Catenin*, *PI3K*, *IL-6*, *TGF-α*, *Bcl2*, *TLR4*, *CEA*, *TGF-β*, *Gli2*, *HES1*,* PTEN*, and *caspase-9* was assayed using the real-time PCR method (Applied Biosystems, USA). The reaction mixture was prepared in a final volume of 20 µL consisting of 1 µL of cDNA, 0.1µM of reverse primer, 0.1µM of forward primer, 5 µL of RNase-free water, and 10 µL of master mix of SYBR Green (Amplicon, Denmark). The sequence of the primers utilized in this study is delineated in Table 1. The internal control was the GAPDH (Glutaldehyde-3-Phosphate Dehydrogenase) gene, and the calibrator was untreated cells. Ultimately, the fold changes in gene expression were determined using standard 2^−ΔΔCT^ calculations (Table 2). The ΔCT and ΔΔCT were calculated by following the formula [35].

$$\Delta CT\,=\,CT\,of\,target\,gene\,-\,CT\,of\,calibrator$$

$$\Delta \Delta CT\,=\,\Delta CT\,of\,target\,gene\, - \,\Delta CT\,of\,calibrator$$

$$Fold\,change\,=\,{2^{ - \Delta \Delta CT}}$$

**Table 1:** The primer sequences were used in this study for real-time PCR method

Oncogenes/ Suppressors	Primers		
	Primer-F	Primer-*R*	reference
*Bcl2*	TGGAGAGTGCTGAAGATTGA	GTCTACTTCCTCTGTGATGTTGTAT	[50]
*β-Catenin*	CTGCTGTTTTGTTCCGAATGTC	CCATTGGCTCTGTTCTGAAGAGA	[51]
*IL-6*	GTCAACTCCATCTGCCCTTCAG	GGTCTGTTGTGGGTGGTATCCT	[52]
*PI3K*	TTGTCTGTCACACTTCTGTAGTT	AACAGTTCCCATTGGATTCAACA	[53]
*TGF-α*	ATGGTCCCCTCGGCTGGACAG	GACCACTGTTTCTGAGTGGCA	[54]
*TGF-β*	CCCAGCATCTGCAAAGCTC	GTCAATGTACAGCTGCCGCA	[55]
*CEA*	TTACCTTTCGGGAGCGAACC	TGTTGCTGCGGTATCCCATT	[56]
*TLR4*	TTTCCTGCAATGGATCAAGGA	TTATCTGAAGGTGTTGCACATTCC	[57]
*Gli2*	AAGTCACTCAAGATTCCTGCTCA	GTTTTCCAGGATGGAGCCACTT	[58]
*HES1*	TCAACACGACACCGGATAAAC	GCCGCGAGCTATCTTTCTTCA	[59]
*PTEN*	CAAGATGATGTTTGAAACTATTCCAATG	CCTTTAGCTGGCAGACCACAA	[60]
*caspase-9*	GCAGGCTCTGGATCTCGGC	GCTGCTTGCCTGTTAGTTCGC	[61]

**Table 2:** Expression of genes (fold change) associated with CRC signaling pathways after treatment. No significant change in expression was showed by Ns

Oncogenes	Fold change			
	CSNP	L.a-sup		CSNP/L.a-sup
*Β-Catenin*	0.07	0.82		0.42
*PI3K*	0.28	1.93		1.07
*IL-6*	0.08	6.39		1.46
*TGF-α*	0.76	0.97 (ns)		0.79
*Bcl2*	0.82 (ns)	11.54		15.4
*TLR4*	0.93 (ns)	3.29		6.40
*CEA*	1.07 (ns)	3.34		10.46
*TGF-β*	0.70	0.22		0.16
*Gli2*	4.91	3.51		11.27
*HES1*	18.6	7.46		40.29
**Suppressors**	**Fold change**			
	**CSNP**	**L.a-sup**	**CSNP/L.a-sup**	
*PTEN*	1.15 (ns)	3.06	42.1	
*caspase-9*	19.49	43.51	114.3	

### Statistical analysis

The analysis of the data was conducted using GraphPad Prism version 8, incorporating the one-way ANOVA and Tukey’s comparison test. A p-value less than 0.05 was considered as significant. The results were evaluated as the mean ± standard deviation (SD). It is noteworthy that all tests were performed in triplicate.

## Results

### Morphological properties of CSNP/L.a-sup

**Fig. 1:**
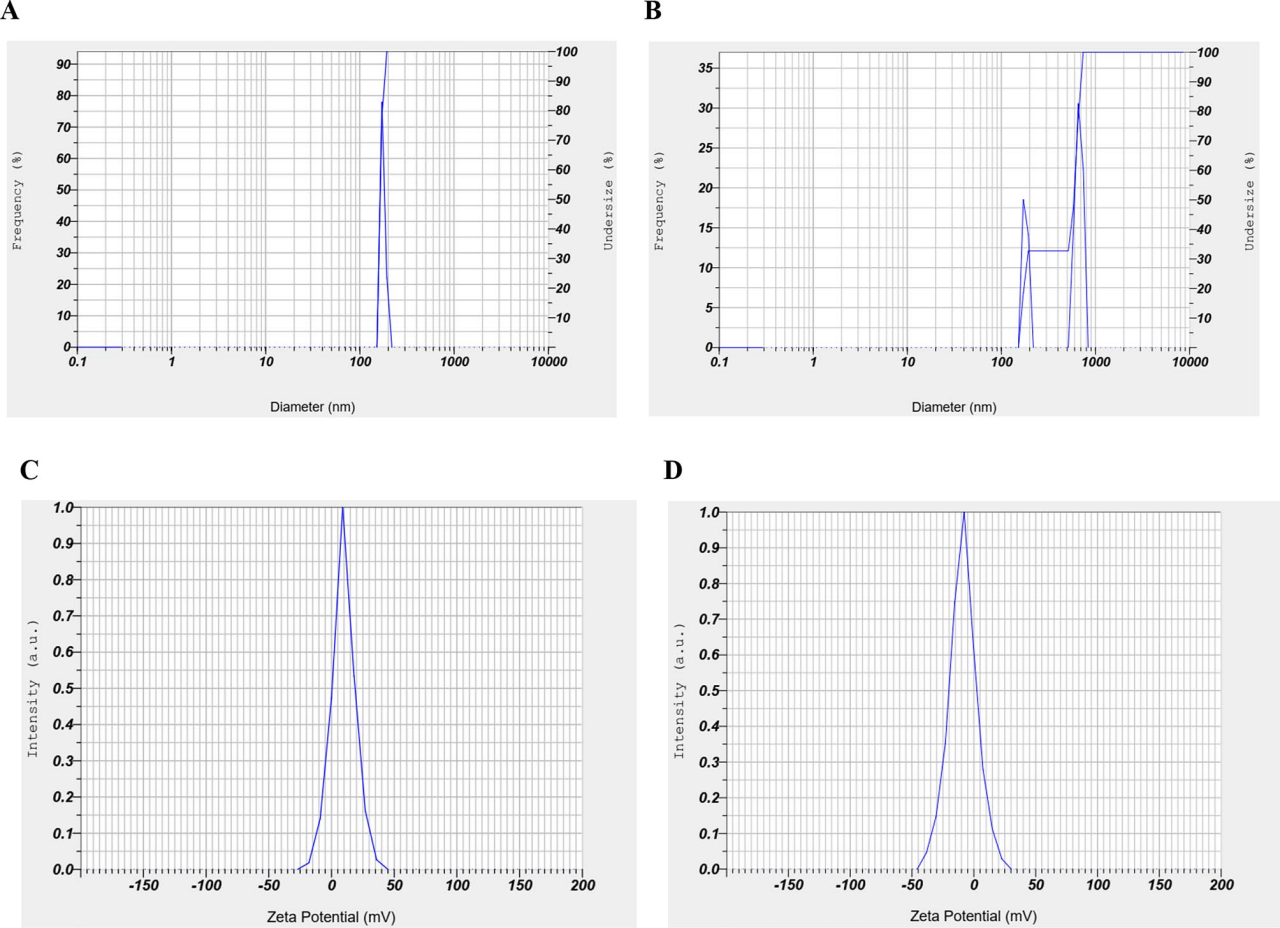
Characterization of CSNP and CSNP/L.a-sup using DLS method. Size distribution of CSNP (**A**) and CSNP/L.a-sup (**B**). Surfaces charge of CSNP (**C**) and CSNP/L.a-sup (**D**)

**Fig. 2:**
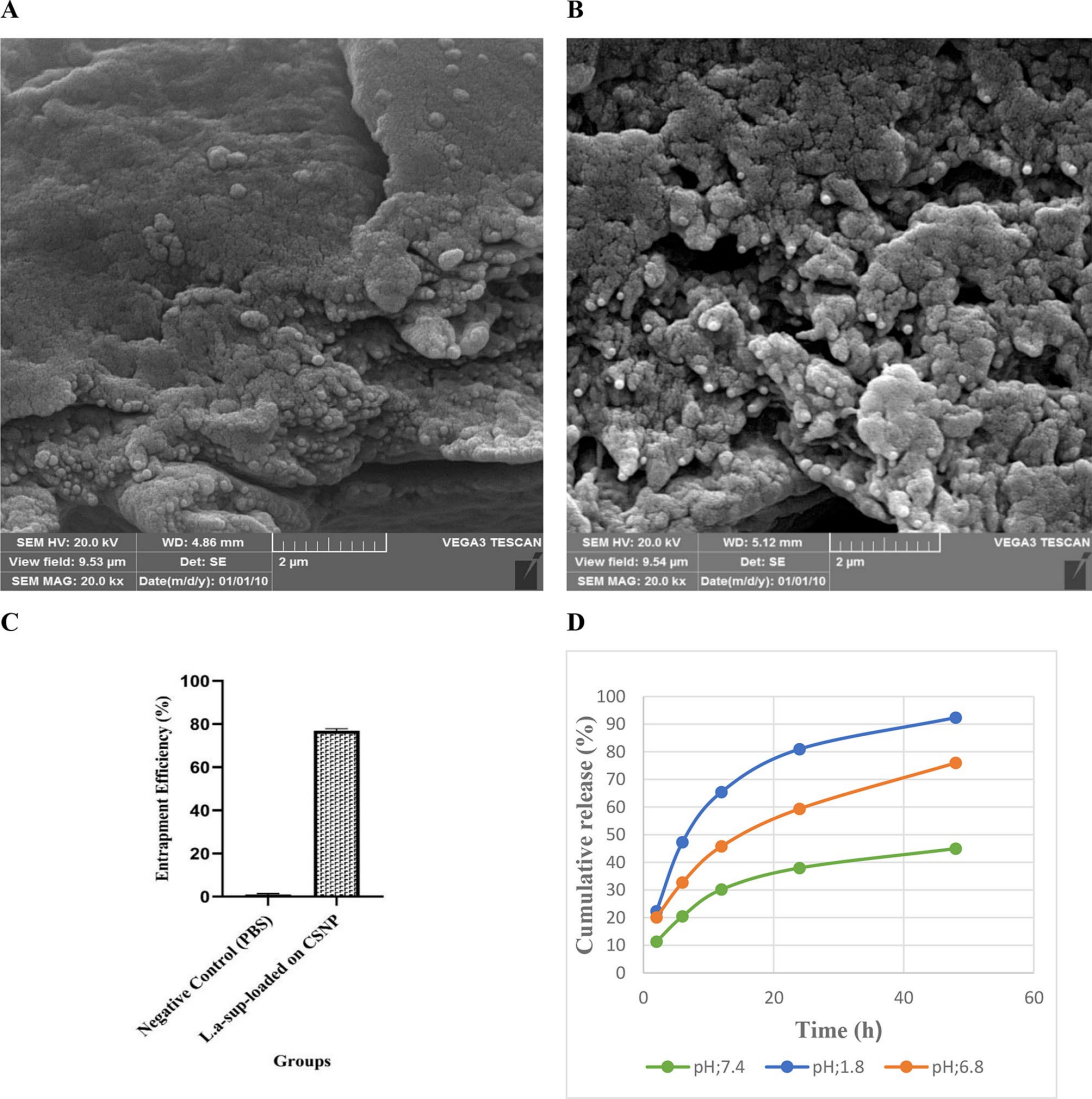
SEM images of CSNP (**A**) and CSNP/L.a-sup (**B**). Entrapment efficiency (EE) of CSNP/L.a-sup (**C**). In-vitro L.a-sup release profile from CSNP/L.a-sup at (blue curve; pH ~ 1.8), (red curve; pH ~ 6.8), and (green curve; pH ~ 7.4) for 48 h, illustrating sustained and controlled release of L.a-sup (**D**)

As demonstrated in Fig. 1A and B, the size of CSNP and CSNP/L.a-sup were 165.7 ± 8.7 and 478.6 ± 219.9 nm, respectively. Furthermore, the histogram images reveal that the surface charge of CSNP and CSNP/L.a-sup were 9.5 and − 8.9 mV, respectively (see Fig. 1C and D) (Table 3). The morphology of the surface of both CSNP and CSNP/L.a-sup is demonstrated in Fig. 2A and B.

**Table 3:** The morphological properties of CSNP and CSNP/L.a-sup

Compound	Chitosan concentration (%)	Nanoparticle size (nm, m ± SD)	Zeta potential (mV)		EE (%)
CSNP	0.05	165.7 ± 8.7		9.5	-
CSNP/L.a-sup	0.05	478.6 ± 219.9		-8.9	74.6

### L.a-sup loaded within CSNP/L.a-sup and EE %

The initial protein content of L.a-sup was determined to be 1340 µg, while the unloaded protein fraction following the formation of CSNP/L.a-sup was found to be 340 µg. Consequently, the loaded protein was calculated to be 1000 µg. As shown in Fig. 2C, EE of CSNP/L.a-sup was calculated equivalent to 74.6%. Therefore, a high amount of L.a-sup was successfully conjugated with CSNP in the new formulation of CSNP/L.a-sup. To ensure a valid comparison, L.a-sup (1340 µg) was diluted with PBS to achieve a final concentration of 1000 µg. Finally, the specified concentrations of CSNP (0.05%), L.a sup (1000 µg), and CSNP/L.a-sup (1000 µg ± 0.05%) with a final volume of 100 µL were utilized for exposure of the cells.

**The amount of the release of protein from CSNP/L.a-sup at a duration time of 48 h**

As demonstrated in Fig. 2D, the release of protein was evaluated at time intervals of 2, 6, 12, 24, and 48 h under three distinct pH values, including 7.4, 6.8, and 1.8. The release of protein from CSNP/L.a-sup at pH ~ 7.4 (intestine-like conditions) was 11.3, 20.4, 30.2, 38, and 45%, respectively. At pH ~ 6.8 (colon-like conditions), it was 20.1, 32.7, and 40%. 5.8, 59.4, and 76%, respectively. At pH ~ 1.8 (stomach-like conditions), the release was 22.4, 47.3, 65.5, 81, and 92.4% at 2, 6, 12, 24, and 48 h time intervals, respectively. The results indicated that the release of protein from CSNP/L.a-sup fluctuates at different pH levels. According to the data, the percentage of protein release at pH 6.8 (colon-like conditions) is optimal, and the highest rate of protein release from CSNP/L.a-sup was detected after 48 h.

### Determination of cellular uptake

**Fig. 3:**
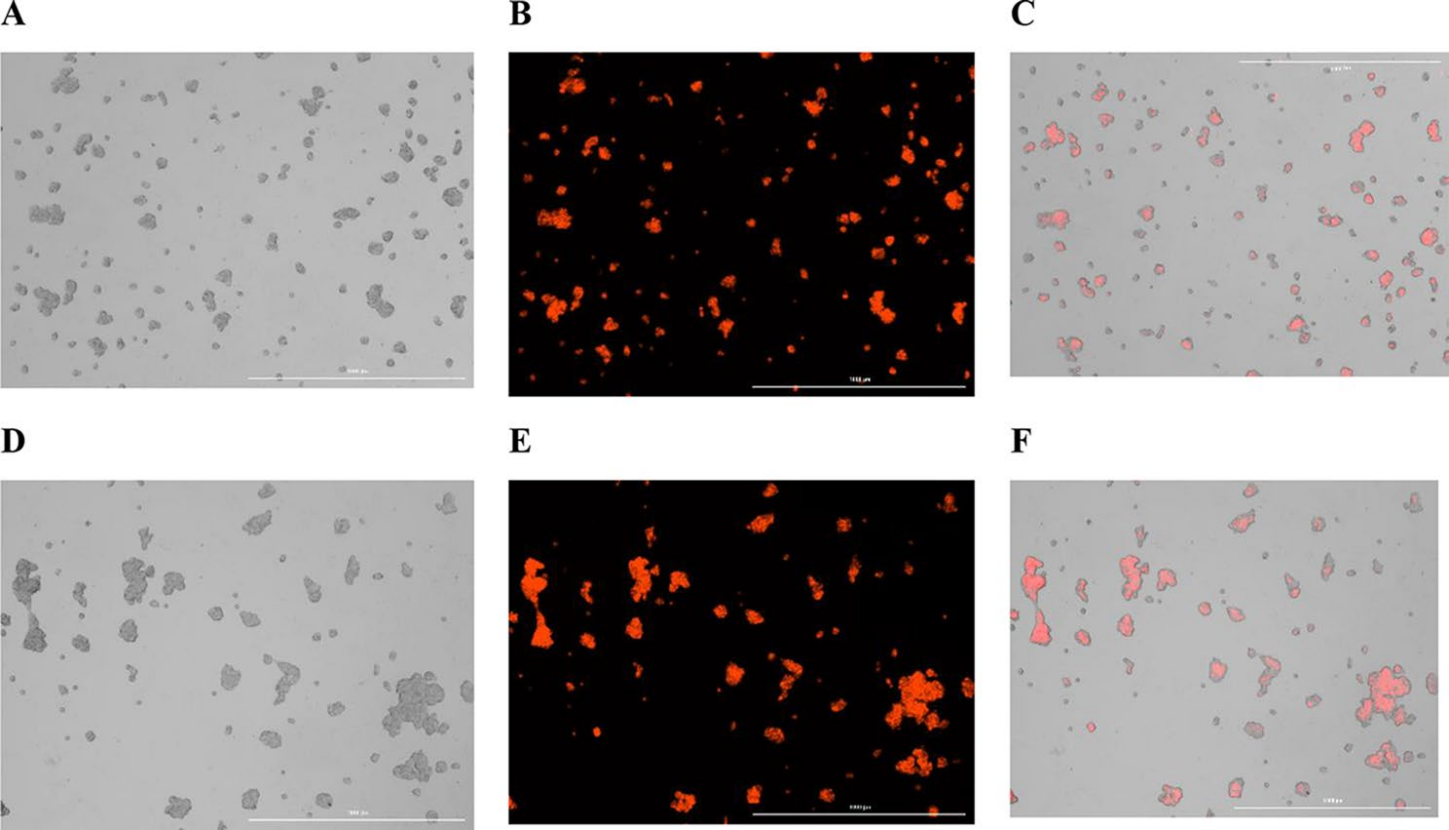
Rhodamine-B labeled Caco-2 cells for cellular uptake. CSNP/L.a-sup examined by bright (**A**), fluorescence (**B**), and merge (**C**) images of CSNP/L.a-sup after 1 h, and bright (**D**), fluorescence (**E**), merge (**F**) images of CSNP/L.a-sup after 2 h

Intracellular uptake is a critical step in evaluating the potential of designed nanoparticles for cancer therapy. To assess the accumulation of CSNP/L.a-sup within cells, the uptake of CSNP/L.a-sup into Caco-2 cells was analyzed. As shown in Fig. 3A-F, the uptake of CSNP/L.a-sup by Caco-2 cells occurs in a time-dependent manner, with initial absorption observed within 1 h and substantial internalization achieved after 2 h.

### Viability of Caco-2 and HDF cells after interaction with CSNP/L.a-sup

**Fig. 4:**
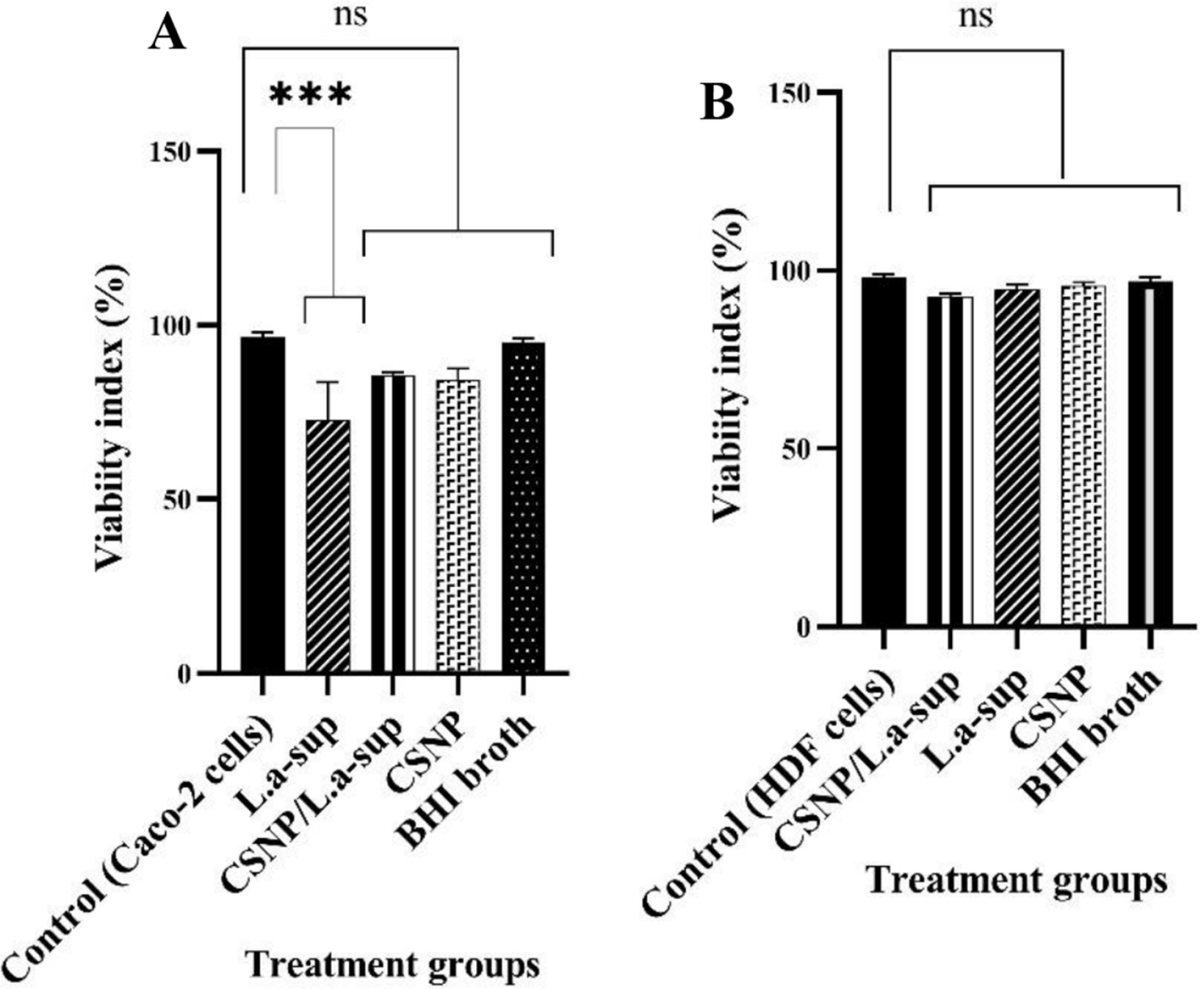
Caco-2 (**A**) and HDF (**B**) cells viability after exposure to CSNP/L.a-sup for 24 h

As demonstrated in Fig. 4A, following the interaction of Caco-2 cells with CSNP (0.05%), L.a Sup (1000 µg), CSNP/L.a-sup (1000 µg + 0.05%), and BHI broth medium (as a control), the cell viability was 84.3%, 72.6%, 85.5%, and 95.2%, respectively. As demonstrated in Fig. 4B, following the interaction of HDF (as normal cells) with CSNP (0.05%), L.a Sup (1000 µg), CSNP/L.a-sup (1000 µg + 0.05%), and BHI broth medium, the percentage of viable cells was 95.7, 94.5, 92.6, and 97%, respectively. The viability of both Caco-2 and HDF cells was found to be above 50% after exposure to each test compound. Furthermore, a comparison of the stability of the HDF cells and Caco-2 cells revealed that the former demonstrated greater stability.

### Regulatory effect of CSNP/L.a-sup on the expression of genes related to CRC signaling pathways

**Fig. 5:**
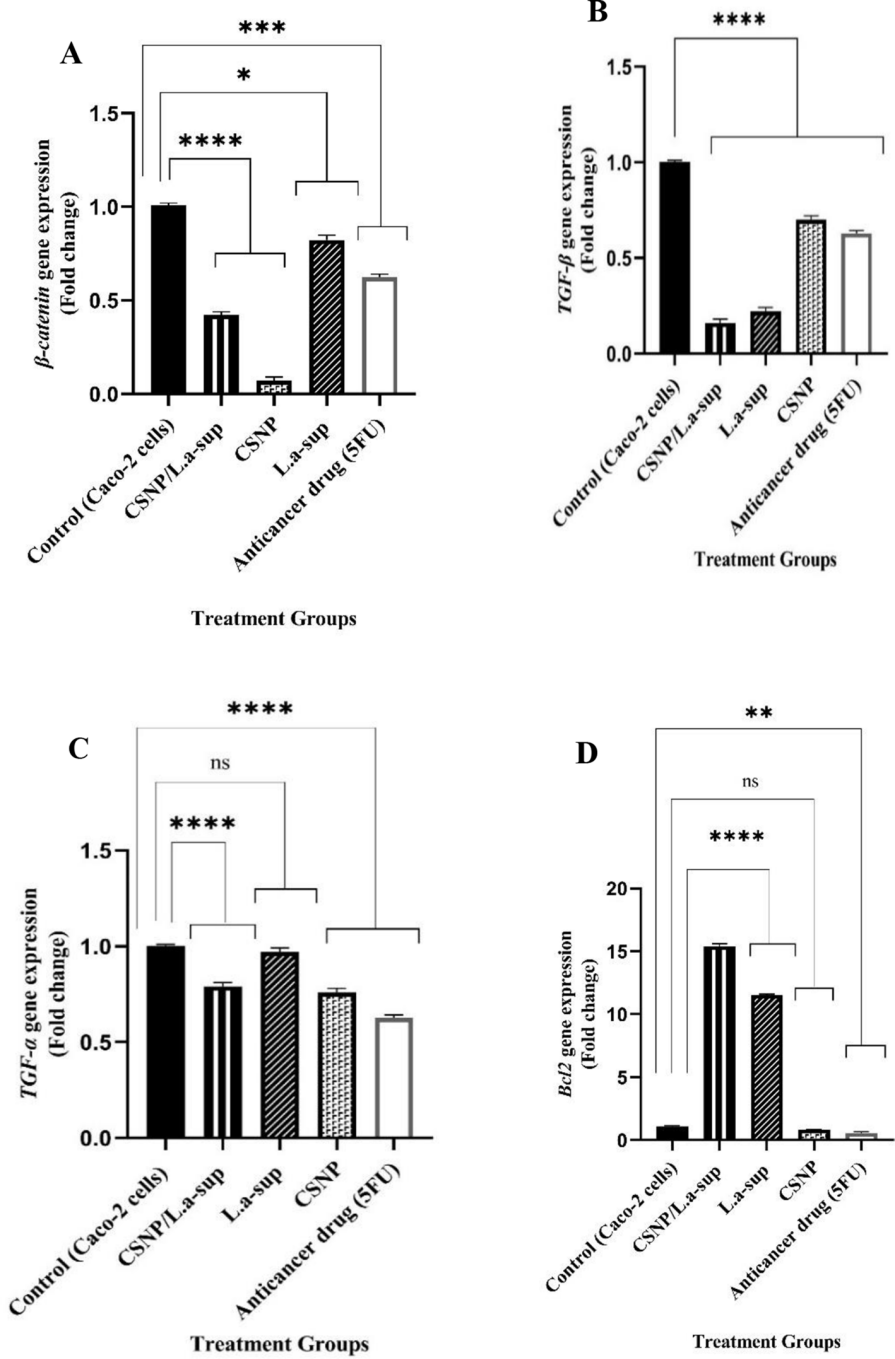
The fold changes in expression of genes related to CRC signaling pathways in Caco-2 cells after exposure with L.a-sup, CSNP, and CSNP/L.a-sup using real-time PCR, representing *β-Catenin* (**A**), *TGF-β* (**B**), *TGF-α* (**C**), *Bcl2* (**D**). Values are presented as the mean ± SD of triplicate independent tests, *p <.0156, **p <.0018, ***p <.0003, and ****p <.0001 indicate statistically significant

**Fig. 6:**
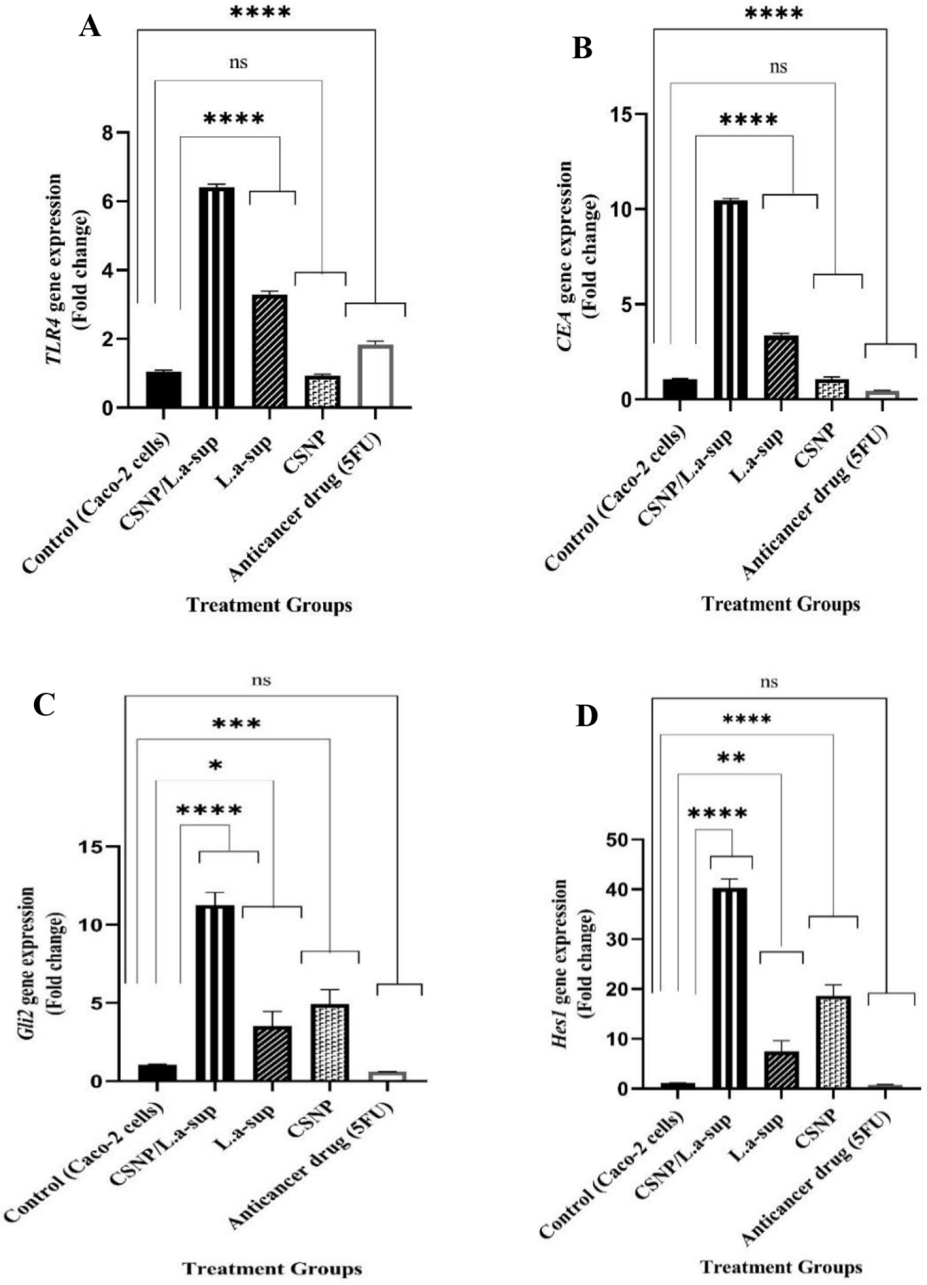
The fold changes in expression of genes related to CRC signaling pathways in Caco-2 cells after exposure with L.a-sup, CSNP, and CSNP/L.a-sup using real-time PCR, representing *TLR4* (**A**), *CEA* (**B**) *Gli2* (**C**), and *HES1* (**D**). Values are presented as the mean ± SD of triplicate independent tests. * *p* <.0101, ***p <.0003, and ****p <.0001 indicate statistically significant

**Fig. 7:**
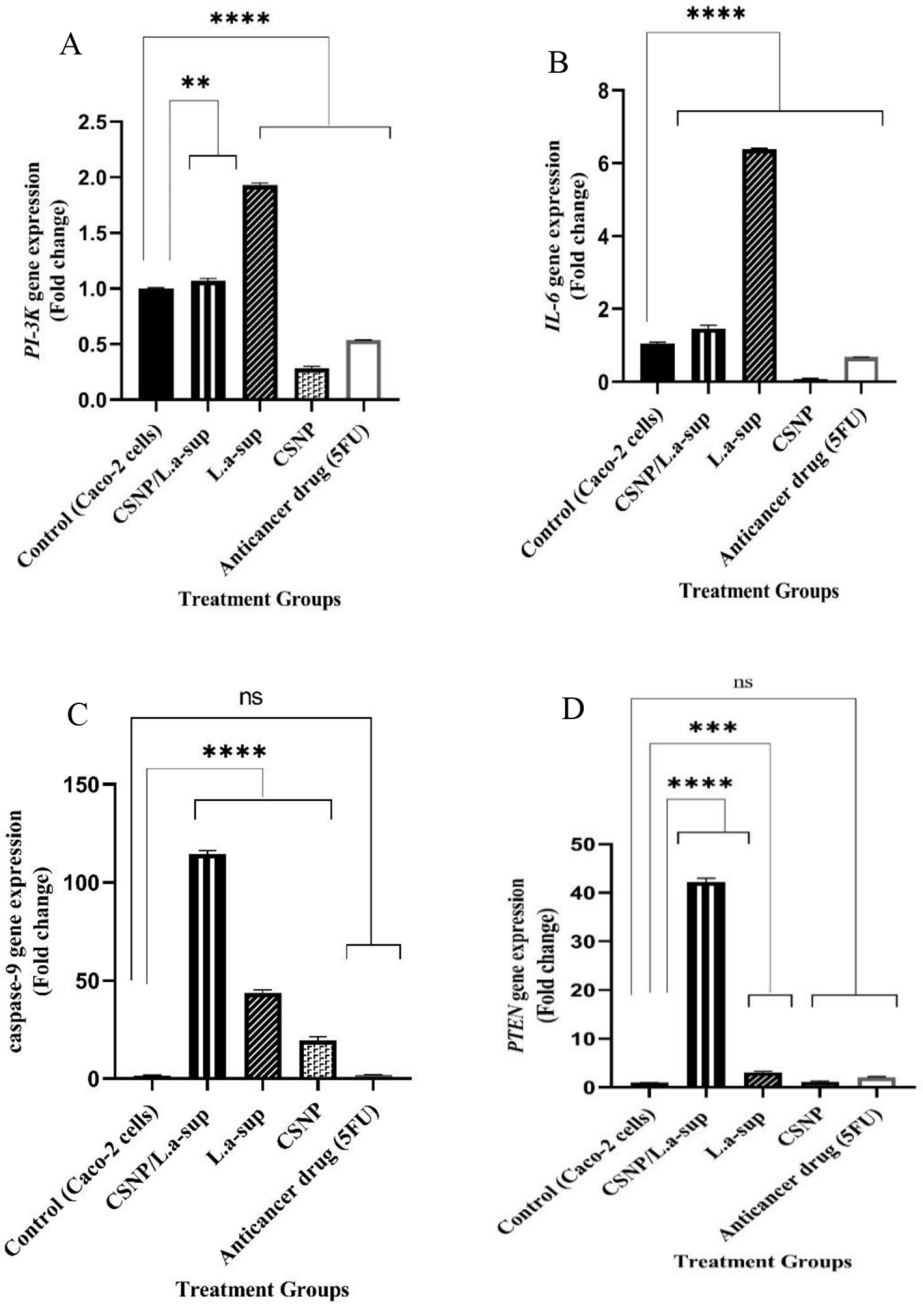
The fold changes in expression of genes related to CRC signaling pathways in Caco-2 cells after exposure with L.a-sup, CSNP, and CSNP/L.a-sup using real-time PCR, representing *PI3K* (**A**), *IL-6* (**B**), *caspase-9* (**C**), and *PTEN* (**D**). Values are presented as the mean ± SD of triplicate independent tests. **p <.0030, ***p <.0007 and ****p <.0001 indicate statistically significant

As demonstrated in Fig. 5A-C, following the interaction of Caco-2 cells with CSNP/L.a-sup, the expression levels of oncogenes of *β-Catenin*, *TGF-α*, and *TGF-β* underwent a significant decrease with a 0.42, 0.79, and 0.16-fold change, respectively (*p* <.0001). Conversely, a substantial augmentation in the expression of *Bcl2* (Fig. 5D), *TLR4*, *CEA*, *Gli2*, *HES1* (Fig. 6A-D), *PI3K*, and *IL-6* oncogenes (Fig. 7A and B) was observed (*p* <.05). As illustrated in Fig. 5C, the CSNP treatment led to a reduction in the expression of the *TGF-α* oncogene, while L.a-sup demonstrated no effect on it. This finding indicates that the CSNP/L.a-sup combination likely exerts its antineoplastic effect through its CSNP compartment. The findings demonstrated that 5-FU can cause a significant decrease in level of *β-Catenin*, *TGF- α*, *TGF-β*, *Bcl2*, *TLR4*, *CEA*, *PI3K*, and *IL-6* genes compared to the control group (*p* <.05), whereas it showed no significant effect on *Gli2*, *HES1*,* caspase-9*, and *PTEN* genes expression compared to CSNP/L.a-sup which caused a remarkable increase in the levels of two suppressors of *caspase-9* and *PTEN* (114.39 and 42.19-fold) compared to 5-FU (2 and 1.95-fold). As demonstrated in Fig. 7A and B, CSNP resulted in a substantial decrease in the expression of *PI3K* and *IL-6* oncogenes (*p* <.0001), while both L.a-sup and CSNP/L.a-sup led to a significant increase in the expression of these genes (*p* <.0001). The findings suggest that probably the suppressive effect of CSNP on the expression of *PI3K* and *IL-6* genes is counterbalanced by the novel formulation of CSNP/L.a-sup.

### Regulatory effect of CSNP/L.a-sup on the expression of suppressor genes associated to CRC signaling pathways

As demonstrated in Fig. 7C, following the interaction of Caco-2 cells with CSNP, L.a Sup, and CSNP/L.a-sup, a significant increase was observed in the expression of the *caspase-9* gene (*p* <.0001). Furthermore, CSNP/L.a-sup significantly increased the expression of both *PTEN* and *caspase-9* suppressors by 42.1- and 114.3-fold, respectively. Conversely, as illustrated in Fig. 7D, the interaction of Caco-2 cells with CSNP exhibited no substantial impact on *PTEN* gene expression, while the expression of the *caspase-9* suppressor gene was augmented. The resultant data unequivocally demonstrate the synergistic effect of CSNP and L.a Sup in CSNP/L.a-Sup, which results in a substantial increase in the expression of suppressor genes associated with CRC signaling pathways.

### Superior efficacy of CSNP/L.a-sup in modulating of the genes associated with CRC signaling pathways

Our data indicated that CSNP has greater efficacy compared to CSNP/L.a-sup and L.a-sup in suppressing the expression of the oncogenes of *B-Catenin*, *PI3K*, *IL-6*, *TGF-α*, and *TGF-β*. Furthermore, CSNP was not able to increase the expression of *PTEN* suppressor gene, while it was able to increase the expression of *caspase-9* gene. However, CSNP/L.a-sup showed a superior efficiency to increase the expression of *PTEN* and *caspase-9* suppressor genes compared to CSNP and L.a-sup.

## Discussion

CRC has become a global concern due to its high mortality rate [36]. Standard cancer treatments are not only difficult and painful, but also have a number of side effects [37]. Scientists are therefore exploring alternative approaches to cancer treatment. In recent years, biotherapy using probiotics [38] and nanotherapy [21] have emerged as new strategies to fight cancer. Several studies have shown that L.a-sup and CSNP have multiple anticancer effects, including anti-proliferative, anti-necrosis, anti-apoptotic, and anti-metastatic properties [39, 40]. The study investigated the effects of L.a-sup encapsulated with CSNP as a drug delivery system on CRC signaling pathway genes under in vitro conditions. In a comprehensive review of chitosan toxicity, the results indicated that CSNP typically has low cytotoxicity in various cell lines. However, it is recommended that any chitosan formulation undergoes a thorough cytotoxicity evaluation prior to any experiment [41]. The current results indicate that the CSNP/L.a-sup formulation has no significant toxic effect on Caco-2 cells. On the other hand, the maximum rate of protein release from CSNP/L.a-sup occurs at pH ~ 6.8, which is similar to the pH of the colon. Therefore, it has been shown that CSNP/L.a-sup is an effective drug delivery system for CRC treatment. The DLS results indicated that 33% of the sample consists of CSNP alone, while 67% of the sample consists of L.a-sup loaded on CSNP. It is conceivable that impurities are invariably present; however, the quantity of CSNP/L.a-sup (67%) is a substantial percentage. Moreover, EE experiment showed that not all of L.a-sup was loaded on CSNP. The amount of L.a-sup loaded on CSNP was 76.9%, and DLS results are consistent with EE% results.

According to our results, the interaction of Caco-2 cells with CSNP, L.a-sup and CSNP/L.a-sup had different effects on the expression of genes related to the signaling pathways of CRC. The present study showed that CSNP/L.a-sup led to a significant decrease in the expression of the oncogenes *β-catenin*, *TGF-α* and *TGF-β*, while it caused a significant increase in the expression of the oncogenes *HES1*, *Gli2*, *CEA*, *PI3K*, *IL-6*, *Bcl2* and *TLR4*. Previous studies have shown that CSNP conjugated with different compounds has different effects on cancer-associated signaling pathways. For example, CSNP conjugated with alginate hydrogel [42], carboxymethyl and oligosaccharides resulted in inhibition of *β-catenin*, *TGF-α* and *TGF-β* expression. A study by Ghanavati R et al. (2020) demonstrated that *Lactobacillus* species could reduce *β-catenin* gene expression, which plays an essential role in CRC progression [43].

Furthermore, CSNP exhibited a superior inhibitory efficiency on the expression of five oncogenes *PI3K*, *IL-6*, *β-Catenin*, *TGF-α*, and *TGF-β* in comparison to CSNP/L.a-sup and L.a-sup. CSNP possesses a smaller size and higher positive charge in comparison to CSNP/L.a-sup and L.a-sup. Consequently, CSNP is absorbed more easily by cells than CSNP/L.a-sup and L.a-sup, although the size and charge of nanoparticles are not the only significant factors affecting cellular uptake. Essential parameters influencing cellular uptake include the type of particle, size distribution, shape, electrical charge, hydrophobicity, polydispersity index, and zeta potential [44]. In a study by Miatmoko A et al., it was reported that the cationic polymer chitosan, which possesses a positive charge, could result in a substantial increase in cellular uptake. The cationic polymer significantly enhances cellular absorption in vitro through electrostatic interactions [45]. As illustrated in Fig. 5A, the expression of *β-Catenin* in the CSNP group is notably lower compared to the CSNP/L.a-sup group. A study by He C et al. (2010) revealed that nanoparticles with a positive charge and an approximate particle size of 150 nm exhibited an increased tendency to accumulate in tumors, suggesting that optimizing particle size and surface charge is crucial in designing drug nanocarriers to maximize therapeutic efficacy [46]. The literature on this subject is inconclusive; some studies suggest that nanospheres adhere to cells less effectively than nanorods, while others indicate the contrary. For instance, in a study by Je HyunJeong JH et al. (2017), it was demonstrated that in Caco-2 cells, cellular uptake of CSNP conjugated with different compounds significantly increased with particle sizes from 200 to 600 nm but declined sharply for sizes above 600 nm [47]. The present study lends further support to the hypothesis advanced in a previous study, which suggested that the enhanced efficacy of CSNP in the suppression of certain oncogenes may be attributable to its diminished size. A notable finding of the present study was that CSNP led to a decrease in the expression of *PI3K* and *IL-6* oncogenes, while L.a-sup resulted in a significant increase in the expression of these genes. The combination of CSNP and L.a-sup subsequently led to a substantial increase in the expression of these genes. The results indicated that the reducing effects of CSNP were counteracted by the novel formulation of CSNP/L.a-sup on the expression of these genes. However, numerous studies have confirmed that CSNP is a unique compound, which shows different effects on cancer signaling pathways after conjugated with various agents such as programmed death ligand 1, ribonucleotide reductase regulatory subunit M2, insulin like growth factor 1 receptor, folic acid, glycyrrhetinic acid, and herceptin [48, 49]. In the present study, the synergistic effect of CSNP and L.a-sup on certain genes associated with CRC signaling pathways is highlighted, underscoring the potential of CSNP/L.a-sup in the management of CRC.

The 5-FU has been observed to result in a substantial decline in the levels of *β-Catenin*, *TGF-α*, *TGF-β*, *Bcl2*, *TLR4*, *CEA*, *PI3K*, and *IL-6* genes when compared to the control group. Conversely, no significant alterations were detected in the levels of *Gli2*, *HES1*, *caspase-9*, and *PTEN* genes when contrasted with the control group. Furthermore, CSNP/L.a-sup has been observed to induce a substantial augmentation in the levels of two suppressors of *caspase-9* and *PTEN* (114.39 and 42.19-fold), in comparison to the effects observed with 5-FU (2 and 1.95-fold). Consequently, the regulatory effect of 5-FU is achieved through the suppression of various oncogenes. In contrast, CSNP/L.a-sup exerts its effect by both decreasing the oncogenes and enhancing the suppressors’ expression, such as the *caspase-9* and *PTEN* genes.

It is noteworthy that in the present study, CSNP/L.a-sup exhibited a higher efficiency in inducing *caspase-9* and *PTEN* gene expression compared to CSNP and L.a-sup, indicative of a synergistic effect. Previous studies have indicated that CSNP and the strains of Lactobacillus induce apoptosis signaling pathways by upregulating the expression of *caspase-9* and *PTEN* genes, respectively [48, 49]. Consequently, the bacterial secretome utilized in the present study can function as the parent live cells and can be regarded as a safe alternative to live probiotic cells in the management of CRC.

The primary conclusions of the present study are as follows: (i) CSNP/L.a-sup demonstrated high efficiency in reducing the expression of several oncogenes, including *β-Catenin*, *TGF-α*, and *TGF-β*; (ii) CSNP/L.a-sup was unsuccessful in reducing the expression of other oncogenes, namely *Gli2*, *HES1*, *CEA*, *PI3K*, *IL-6*, *Bcl2*, and *TLR4*; (iii) CSNP/L.a-sup was found to be efficacious in increasing the expression of both suppressor genes, *caspase-9* and *PTEN*; (iv) CSNP and L.a-sup were found to demonstrate superior synergistic efficiency in inducing the expression of repressor genes. It is therefore evident that, owing to the optimal release of CSNP/L.a-sup in conditions analogous to the colon, low toxicity on Caco-2 cells, and high efficiency in regulating CRC signaling pathway genes, the nanoparticle exhibited superior properties to combat CRC.

However, it should be noted that this research was subject to certain limitations. It is therefore recommended that further experiments be conducted, such as in vivo anti-tumor efficacy testing of CSNP/L.a-sup, in order to further confirm the anti-cancer effects of CSNP/L.a-sup.

## Conclusion

In conclusion, the present study demonstrates that the designed nanostructure exhibits superior properties for combating CRC, owing to the optimal release of CSNP/L.a-sup in conditions analogous to the colon, low toxicity on Caco-2 cells, and high efficiency in regulating CRC signaling pathway genes. The results suggest that CSNP/L.a-sup could play a significant role in managing CRC progression, and the nanoparticle is recommended as a promising biotherapeutic and nanotherapeutic approach for managing CRC. However, further investigation is required to fully explore its potential clinical applications in vivo.

## Data Availability

Data is provided within the manuscript.

## Abbreviations

CRC: Colorectal cancer
CSNP/L.a-sup: Chitosan nanoparticle conjugated with lactobacillus acidophilus secretome
EE: Encapsulation efficiency
Caco-2: Colon adenocarcinoma
HDF: Human dermal fibroblasts cells
PCR: Polymerase chain reaction
PI3K: Phosphatidylinositol 3-kinase
TGFβ: Transforming growth factor
EGFR: Epidermal growth factor receptor
SHH: Hedgehog pathway
IL-6: Interleukin 6
TLR4: Toll-like receptor 4
CEA: Carcinoembryonic antigen
L. acidophilus: Lactobacillus acidophilus
5-FU: 5-fluorouracil
CFS: Cell free supernatant
MRS agar: de man Rogosa and Sharpe agar
BHI: Brain heart infusion
OD: Optical density
PBS: Phosphate-buffered saline
TPP: Tripolyphosphate
DLS: Dynamic light scattering
ZP: Zeta potential
SEM: Scanning electron microscopy
Rh B: Rhodamine B
DMEM: Dulbecco’s Modified Eagle’s Medium
FBS: Fetal bovin serum
DMSO: Dimethyl sulfoxide
cDNA: Complementary DNA
GAPDH: Glutaldehyde3-phosphate dehydrogenase

## References

[1] Koveitypour Z, Panahi F, Vakilian M, Peymani M, Seyed Forootan F, Nasr Esfahani MH, Ghaedi K. Signaling pathways involved in colorectal cancer progression. Cell Bioscience. 2019;9(1):97. 31827763 10.1186/s13578-019-0361-4 PMC6889432

[2] Akimoto N, Ugai T, Zhong R, Hamada T, Fujiyoshi K, Giannakis M, et al. Rising incidence of early-onset colorectal cancer—A call to action. Nat Reviews Clin Oncol. 2021;18(4):230–43. 10.1038/s41571-020-00445-1 PMC7994182 33219329

[3] Roshandel G, Ferlay J, Ghanbari-Motlagh A, Partovipour E, Salavati F, Aryan K, et al. Cancer in Iran 2008 to 2025: recent incidence trends and short‐term predictions of the future burden. Int J Cancer. 2021;149(3):594–605. 33884608 10.1002/ijc.33574

[4] Alzahrani SM, Al Doghaither HA, Al-Ghafari AB. General insight into cancer: an overview of colorectal cancer. Mol Clin Oncol. 2021;15(6):271. 34790355 10.3892/mco.2021.2433 PMC8591689

[5] Rahmati A, Homayouni Tabrizi M, Karimi E, Zarei B. Fabrication and assessment of folic acid conjugated-chitosan modified PLGA nanoparticle for delivery of alpha Terpineol in colon cancer. J Biomater Sci Polym Ed. 2022;33(10):1289–307. 35260045 10.1080/09205063.2022.2051693

[6] Luo H, Su H, Wang X, Wang L, Li J. N-Succinyl-chitosan nanoparticles induced mitochondria-dependent apoptosis in K562. Mol Cell Probes. 2012;26(4):164–9. 22465598 10.1016/j.mcp.2012.03.006

[7] Tincer G, Bayyurt Kocabaş B, Arica YM, Gursel I. Chitosan polysaccharide suppress toll like receptor dependent immune response. 2015.

[8] Huang I-F, Lin I-C, Liu P-F, Cheng M-F, Liu Y-C, Hsieh Y-D, et al. Lactobacillus acidophilus attenuates Salmonella-induced intestinal inflammation via TGF-β signaling. BMC Microbiol. 2015;15:1–9. 26446848 10.1186/s12866-015-0546-x PMC4596496

[9] Bian J, Dannappel M, Wan C, Firestein R. Transcriptional regulation of Wnt/β-catenin pathway in colorectal cancer. Cells. 2020;9(9):2125. 32961708 10.3390/cells9092125 PMC7564852

[10] Ruman U, Buskaran K, Pastorin G, Masarudin MJ, Fakurazi S, Hussein MZ. Synthesis and characterization of chitosan-based nanodelivery systems to enhance the anticancer effect of Sorafenib drug in hepatocellular carcinoma and colorectal adenocarcinoma cells. Nanomaterials. 2021;11(2):497. 33669332 10.3390/nano11020497 PMC7920308

[11] Tang G, Zhang L. Update on strategies of probiotics for the prevention and treatment of colorectal cancer. Nutr Cancer. 2022;74(1):27–38. 33356609 10.1080/01635581.2020.1865420

[12] Rashedi J, Haghjo AG, Abbasi MM, Tabrizi AD, Yaqoubi S, Sanajou D, et al. Anti-tumor effect of Quercetin loaded Chitosan nanoparticles on induced colon cancer in Wistar rats. Adv Pharm Bull. 2019;9(3):409. 31592135 10.15171/apb.2019.048 PMC6773937

[13] Rudzinski WE, Palacios A, Ahmed A, Lane MA, Aminabhavi TM. Targeted delivery of small interfering RNA to colon cancer cells using Chitosan and pegylated Chitosan nanoparticles. Carbohydr Polym. 2016;147:323–32. 27178938 10.1016/j.carbpol.2016.04.041

[14] Thantsha M, Mamvura C, Booyens J. Probiotics–what they are, their benefits and challenges. New Advances in the Basic and Clinical Gastroenterology. 2012;21.

[15] Isazadeh A, Hajazimian S, Shadman B, Safaei S, Bedoustani AB, Chavoshi R, et al. Anti-cancer effects of probiotic lactobacillus acidophilus for colorectal cancer cell line caco-2 through apoptosis induction. Pharm Sci. 2020;27(2):262–7.

[16] An J, Ha E-M. Combination therapy of Lactobacillus plantarum supernatant and 5-fluouracil increases chemosensitivity in colorectal cancer cells. J Microbiol Biotechnol. 2016;26(8):1490–503. 27221111 10.4014/jmb.1605.05024

[17] Nataraj BH, Ali SA, Behare PV, Yadav H. Postbiotics-parabiotics: the new horizons in microbial biotherapy and functional foods. Microb Cell Fact. 2020;19:1–22. 32819443 10.1186/s12934-020-01426-w PMC7441679

[18] Sagmeister T, Gubensäk N, Buhlheller C, Grininger C, Eder M, Ðordić A, et al. The molecular architecture of Lactobacillus S-layer: assembly and attachment to teichoic acids. Proc Natl Acad Sci. 2024;121(24):e2401686121. 38838019 10.1073/pnas.2401686121 PMC11181022

[19] Guamán LP, Carrera-Pacheco SE, Zúñiga-Miranda J, Teran E, Erazo C, Barba-Ostria C. The impact of bioactive molecules from probiotics on child health: A comprehensive review. Nutrients. 2024;16(21):3706. 39519539 10.3390/nu16213706 PMC11547800

[20] Öztürk K, Kaplan M, Çalış S. Effects of nanoparticle size, shape, and zeta potential on drug delivery. Int J Pharm. 2024:124799. 10.1016/j.ijpharm.2024.124799 39369767

[21] Sachdeva B, Sachdeva P, Negi A, Ghosh S, Han S, Dewanjee S, et al. Chitosan nanoparticles-based cancer drug delivery: application and challenges. Mar Drugs. 2023;21(4):211. 37103352 10.3390/md21040211 PMC10142570

[22] Rajam M, Pulavendran S, Rose C, Mandal A. Chitosan nanoparticles as a dual growth factor delivery system for tissue engineering applications. Int J Pharm. 2011;410(1–2):145–52. 21392563 10.1016/j.ijpharm.2011.02.065

[23] Wu D, Fu K, Zhang W, Li Y, Ji Y, Dai Y, Yang G. Chitosan nanomedicines-engineered bifidobacteria complexes for effective colorectal tumor-targeted delivery of SN-38. Int J Pharm. 2024:124283. 10.1016/j.ijpharm.2024.124283 38810933

[24] Abdel-Hakeem MA, Mongy S, Hassan B, Tantawi OI, Badawy I. Curcumin loaded chitosan-protamine nanoparticles revealed antitumor activity via suppression of NF-κB, Proinflammatory cytokines and Bcl-2 gene expression in the breast cancer cells. J Pharm Sci. 2021;110(9):3298–305. 34097977 10.1016/j.xphs.2021.06.004

[25] Amirani E, Hallajzadeh J, Asemi Z, Mansournia MA, Yousefi B. Effects of Chitosan and oligochitosans on the phosphatidylinositol 3-kinase-AKT pathway in cancer therapy. Int J Biol Macromol. 2020;164:456–67. 32693135 10.1016/j.ijbiomac.2020.07.137

[26] Bahmani S, Azarpira N, Moazamian E. Anti-colon cancer activity of Bifidobacterium metabolites on colon cancer cell line SW742. Turkish J Gastroenterol. 2019;30(9):835. 10.5152/tjg.2019.18451 PMC6750814 31530527

[27] Dallal MMS, Mojarrad M, Baghbani F, Raoofian R, Mardaneh J, Salehipour Z. Effects of probiotic Lactobacillus acidophilus and Lactobacillus casei on colorectal tumor cells activity (CaCo-2). Arch Iran Med. 2015;18(3):0. 25773690

[28] Saberpour M, Najar-Peeraye S, Shams S, Bakhshi B. Effects of Chitosan nanoparticles loaded with mesenchymal stem cell conditioned media on gene expression in Vibrio cholerae and Caco-2 cells. Sci Rep. 2022;12(1):9781. 35697926 10.1038/s41598-022-14057-5 PMC9192724

[29] Honary S, Ebrahimi P, Hadianamrei R. Optimization of size and encapsulation efficiency of 5-FU loaded Chitosan nanoparticles by response surface methodology. Curr Drug Deliv. 2013;10(6):742–52. 24274636 10.2174/15672018113109990049

[30] Loo C-Y, Traini D, Young PM, Parumasivam T, Lee W-H. Pulmonary delivery of Curcumin and Quercetin nanoparticles for lung cancer–Part 1: aerosol performance characterization. J Drug Deliv Sci Technol. 2023;86:104646.

[31] Palanikumar L, Al-Hosani S, Kalmouni M, Nguyen VP, Ali L, Pasricha R, et al. pH-responsive high stability polymeric nanoparticles for targeted delivery of anticancer therapeutics. Commun Biology. 2020;3(1):95. 10.1038/s42003-020-0817-4 PMC7054360 32127636

[32] Liu Z, Li N, Liu P, Qin Z, Jiao T. Highly sensitive detection of iron ions in aqueous solutions using fluorescent Chitosan nanoparticles functionalized by Rhodamine B. ACS Omega. 2022;7(6):5570–7. 35187371 10.1021/acsomega.1c07071 PMC8851898

[33] Mahmoudjanlou H, Saberpour M, Bakhshi B. Antimicrobial, anti-adhesive, and anti-invasive effects of condition media derived from adipose mesenchymal stem cells against Shigella flexneri. Arch Microbiol. 2024;206(4):142. 38441673 10.1007/s00203-024-03860-5

[34] Kowapradit J, Opanasopit P, Ngawhirunpat T, Apirakaramwong A, Rojanarata T, Ruktanonchai U, Sajomsang W. In vitro permeability enhancement in intestinal epithelial cells (Caco-2) monolayer of water soluble quaternary ammonium Chitosan derivatives. AAPS PharmSciTech. 2010;11:497–508. 20333490 10.1208/s12249-010-9399-7 PMC2902307

[35] Yilmaz A, Onen HI, Alp E, Menevse S. Real-time PCR for gene expression analysis. INTECH. 2012 May 30;12:229–54.

[36] Dolatkhah R, Somi MH, Bonyadi MJ, Asvadi Kermani I, Farassati F, Dastgiri S. Colorectal cancer in Iran: molecular epidemiology and screening strategies. J cancer Epidemiol. 2015;2015(1):643020. 25685149 10.1155/2015/643020 PMC4312646

[37] Shinji S, Yamada T, Matsuda A, Sonoda H, Ohta R, Iwai T, et al. Recent advances in the treatment of colorectal cancer: a review. J Nippon Med School. 2022;89(3):246–54. 10.1272/jnms.JNMS.2022_89-310 35082204

[38] Kahouli I, Malhotra M, Westfall S, Alaoui-Jamali MA, Prakash S. Design and validation of an orally administrated active L. fermentum-L. Acidophilus probiotic formulation using colorectal cancer apc min/++ mouse model. Appl Microbiol Biotechnol. 2017;101:1999–2019. 27837314 10.1007/s00253-016-7885-x

[39] Adhikari HS, Yadav PN. Anticancer activity of Chitosan, Chitosan derivatives, and their mechanism of action. Int J Biomaterials. 2018;2018(1):2952085. 10.1155/2018/2952085 PMC6332982 30693034

[40] Tripathy A, Dash J, Kancharla S, Kolli P, Mahajan D, Senapati S, Jena MK. Probiotics: a promising candidate for management of colorectal cancer. Cancers. 2021;13(13):3178. 34202265 10.3390/cancers13133178 PMC8268640

[41] Frigaard J, Jensen JL, Galtung HK, Hiorth M. The potential of Chitosan in nanomedicine: an overview of the cytotoxicity of Chitosan based nanoparticles. Front Pharmacol. 2022;13:880377. 35600854 10.3389/fphar.2022.880377 PMC9115560

[42] Cömez B, Özbaş S. Alginate-Chitosan hydrogels containing ShRNA plasmid for Inhibition of CTNNB1 expression in breast Cancer cells. Gels. 2023;9(7):541. 37504420 10.3390/gels9070541 PMC10378784

[43] Ghanavati R, Asadollahi P, Shapourabadi MB, Razavi S, Talebi M, Rohani M. Inhibitory effects of lactobacilli cocktail on HT-29 colon carcinoma cells growth and modulation of the Notch and Wnt/β-catenin signaling pathways. Microb Pathog. 2020;139:103829. 31682995 10.1016/j.micpath.2019.103829

[44] Moraru C, Mincea M, Menghiu G, Ostafe V. Understanding the factors influencing chitosan-based nanoparticles-protein Corona interaction and drug delivery applications. Molecules. 2020;25(20):4758. 33081296 10.3390/molecules25204758 PMC7587607

[45] Miatmoko A, Hariawan BS, Cahyani DM, Sari R, Dinaryanti A, Hendrianto E. The effect of Chitosan addition on cellular uptake and cytotoxicity of ursolic acid niosomes. An Acad Bras Cienc. 2021;93(3):e20201850. 34287462 10.1590/0001-3765202120201850

[46] He C, Hu Y, Yin L, Tang C, Yin C. Effects of particle size and surface charge on cellular uptake and biodistribution of polymeric nanoparticles. Biomaterials. 2010;31(13):3657–66. 20138662 10.1016/j.biomaterials.2010.01.065

[47] Je HJ, Kim ES, Lee JS, Lee HG. Release properties and cellular uptake in caco-2 cells of size-controlled chitosan nanoparticles. J Agric Food Chem. 2017 Dec 20;65(50):10899–906. 10.1021/acs.jafc.7b03627 29172499

[48] Asoudeh-Fard A, Barzegari A, Dehnad A, Bastani S, Golchin A, Omidi Y. Lactobacillus plantarum induces apoptosis in oral cancer KB cells through upregulation of PTEN and downregulation of MAPK signalling pathways. BioImpacts: BI. 2017;7(3):193. 29159146 10.15171/bi.2017.22 PMC5684510

[49] Abdulmalek SA, Saleh AM, Shahin YR, El Azab EF. Functionalized siRNA-chitosan nanoformulations promote triple-negative breast cancer cell death via blocking the miRNA-21/AKT/ERK signaling axis: in-silico and in vitro studies. Naunyn Schmiedebergs Arch Pharmacol. 2024 Sep;397(9):6941–62. 10.1007/s00210-024-03068-w PMC11422444 38592437

[50] Briton-Jones C, Lok IH, Po ALS, Cheung CK, Chiu TT, Haines C. Changes in the ratio of Bax and Bcl-2 mRNA expression and their cellular localization throughout the ovulatory cycle in the human oviduct. J Assist Reprod Genet. 2006;23:149–56. 16575548 10.1007/s10815-005-9012-2 PMC3455039

[51] Nakamura Y, Nawata M, Wakitani S. Expression profiles and functional analyses of Wnt-related genes in human joint disorders. Am J Pathol. 2005;167(1):97–105. 15972956 10.1016/S0002-9440(10)62957-4 PMC1603448

[52] Waldner MJ, Foersch S, Neurath MF. Interleukin-6-a key regulator of colorectal cancer development. Int J Biol Sci. 2012;8(9):1248. 23136553 10.7150/ijbs.4614 PMC3491448

[53] Riquelme I, Tapia O, Espinoza JA, Leal P, Buchegger K, Sandoval A, et al. The gene expression status of the PI3K/AKT/mTOR pathway in gastric cancer tissues and cell lines. Pathol Oncol Res. 2016;22:797–805. 27156070 10.1007/s12253-016-0066-5 PMC5890336

[54] Baldwin GS, Zhang Q-X. Measurement of Gastrin and transforming growth factor α messenger RNA levels in colonic carcinoma cell lines by quantitative polymerase chain reaction. Cancer Res. 1992;52(8):2261–7. 1559230

[55] Xie S, Macedo P, Hew M, Nassenstein C, Lee K-Y, Chung KF. Expression of transforming growth factor-β (TGF-β) in chronic idiopathic cough. Respir Res. 2009;10:1–10. 19463161 10.1186/1465-9921-10-40 PMC2688489

[56] Khan WN, Frängsmyr L, Teglund S, Israelsson A, Bremer K, Hammarström S. Identification of three new genes and Estimation of the size of the carcinoembryonic antigen family. Genomics. 1992;14(2):384–90. 1427854 10.1016/s0888-7543(05)80230-7

[57] Allhorn S, Böing C, Koch AA, Kimmig R, Gashaw I. TLR3 and TLR4 expression in healthy and diseased human endometrium. Reproductive Biology Endocrinol. 2008;6:1–11. 10.1186/1477-7827-6-40 PMC2543020 18775079

[58] Laurendeau I, Ferrer M, Garrido D, D’Haene N, Ciavarelli P, Basso A, et al. Gene expression profiling of the Hedgehog signaling pathway in human meningiomas. Mol Med. 2010;16:262–70. 20386868 10.2119/molmed.2010.00005 PMC2896461

[59] Liu Z-H, Dai X-M, Du B. Hes1: a key role in stemness, metastasis and multidrug resistance. Cancer Biol Ther. 2015;16(3):353–9. 25781910 10.1080/15384047.2015.1016662 PMC4622741

[60] Shen YH, Zhang L, Gan Y, Wang X, Wang J, LeMaire SA, et al. Up-regulation of PTEN (phosphatase and tensin homolog deleted on chromosome ten) mediates p38 MAPK stress signal-induced Inhibition of insulin signaling: a cross-talk between stress signaling and insulin signaling in resistin-treated human endothelial cells. J Biol Chem. 2006;281(12):7727–36. 16418168 10.1074/jbc.M511105200

[61] Sharifi M, Moridnia A. Apoptosis-inducing and antiproliferative effect by Inhibition of miR-182-5p through the regulation of CASP9 expression in human breast cancer. Cancer Gene Ther. 2017;24(2):75–82. 28084318 10.1038/cgt.2016.79

